# The Endophytic Microbiome as a Hotspot of Synergistic Interactions, with Prospects of Plant Growth Promotion

**DOI:** 10.3390/biology10020101

**Published:** 2021-02-01

**Authors:** Udaya Kumar Vandana, Jina Rajkumari, L. Paikhomba Singha, Lakkakula Satish, Hemasundar Alavilli, Pamidimarri D.V.N. Sudheer, Sushma Chauhan, Rambabu Ratnala, Vanisri Satturu, Pranab Behari Mazumder, Piyush Pandey

**Affiliations:** 1 Department of Biotechnology, Assam University Silchar, Assam 788011, India; uday21microbe@gmail.com (U.K.V.); pbmazumder65@gmail.com (P.B.M.); 2 Department of Microbiology, Assam University Silchar, Assam 788011, India; jinark91@gmail.com (J.R.); paikhom.as.in@gmail.com (L.P.S.); 3 Avram and Stella Goldstein-Goren Department of Biotechnology Engineering and the Ilse Katz Center for Meso and Nanoscale Science and Technology, Ben-Gurion University of the Negev, Beer Sheva 84105, Israel; satishlakkakula85@gmail.com; 4 The Albert Katz International School for Desert Studies, The Jacob Blaustein Institutes for Desert Research, Ben-Gurion University of the Negev, Beer Sheva 84105, Israel; 5 Department of Biochemistry and Molecular Biology, College of Medicine, Korea Molecular Medicine and Nutrition Research Institute, Korea University, Seoul 02841, Korea; alavilli.sundar@gmail.com; 6 Amity Institute of Biotechnology, Amity University Chhattisgarh, Raipur 493225, India; pdvnsudheer@gmail.com (P.D.V.N.S.); sushma.chauhan163@gmail.com (S.C.); 7 TATA Institute for Genetics and Society, Bangalore 560065, India; rambabu.ratnala@gmail.com; 8 Institute of Biotechnology, Professor Jayashankar Telangana State Agricultural University, Rajendranagar, Hyderabad 500030, India; vanisreedhar1994@gmail.com

**Keywords:** root endophyte, rhizo-microbiome, rhizosphere, plant growth promotion, PGPR, plant-bacteria interaction

## Abstract

**Simple Summary:**

Endophytic bacteria are plant-associated bacteria that live in the internal tissues of the plant without harming the host plant. They have an important role in plant growth promotion, as they directly or indirectly promote plant growth. They do it by inhibiting the growth of plant pathogens, and/or by producing various secondary metabolites. They are used in the agricultural sector as an eco-friendly alternative tool that helps to improve crop yield. Detection of plant defense response and identification of compounds synthesized by root endophytes are an effective means for their utilization in the agriculture sector as biofertilizers. Therefore, it is important to study the diversity of root endophytic microbial community, endophyte-host plant interactions and their colonization, and their activity for successful application in agricultural lands. Here, in this review, the potential of the root endophytic microbial community, colonization, and role in the improvement of plant growth has been explained. This could mark the potential use of endophytes for the benefit of plant growth and enhanced yield.

**Abstract:**

The plant root is the primary site of interaction between plants and associated microorganisms and constitutes the main components of plant microbiomes that impact crop production. The endophytic bacteria in the root zone have an important role in plant growth promotion. Diverse microbial communities inhabit plant root tissues, and they directly or indirectly promote plant growth by inhibiting the growth of plant pathogens, producing various secondary metabolites. Mechanisms of plant growth promotion and response of root endophytic microorganisms for their survival and colonization in the host plants are the result of complex plant-microbe interactions. Endophytic microorganisms also assist the host to sustain different biotic and abiotic stresses. Better insights are emerging for the endophyte, such as host plant interactions due to advancements in ‘omic’ technologies, which facilitate the exploration of genes that are responsible for plant tissue colonization. Consequently, this is informative to envisage putative functions and metabolic processes crucial for endophytic adaptations. Detection of cell signaling molecules between host plants and identification of compounds synthesized by root endophytes are effective means for their utilization in the agriculture sector as biofertilizers. In addition, it is interesting that the endophytic microorganism colonization impacts the relative abundance of indigenous microbial communities and suppresses the deleterious microorganisms in plant tissues. Natural products released by endophytes act as biocontrol agents and inhibit pathogen growth. The symbiosis of endophytic bacteria and arbuscular mycorrhizal fungi (AMF) affects plant symbiotic signaling pathways and root colonization patterns and phytohormone synthesis. In this review, the potential of the root endophytic community, colonization, and role in the improvement of plant growth has been explained in the light of intricate plant-microbe interactions.

## 1. Introduction

The need for food security is becoming increasingly urgent, as it is anticipated that the worldwide population, which is currently at around 7 billion, will almost increase to 10 billion or more in the following 50 years [1]. The arable land for farming is turning to be a limited resource because of the urban turn of events and industrialization; henceforth, existing agricultural land should be used more efficiently, utilizing suitable agricultural practices [2]. The excessive use of synthetic fertilizer and other agrochemicals is increasing to achieve higher yield. However, this is not a sustainable strategy, because of the negative impacts it imposes on the environment. Soil health is affected through inappropriate fertilizer usage and also by continuous monoculture cropping and pesticide use (rhizosphere auto-toxicity) [3]. Besides, seepage of water from agrochemical-treated fields into water-bodies prompts eutrophication that affects the aquatic ecosystems [4].

Hence, researchers are focused on a wide range of opportunities to attain efficient agricultural land usage. The bacteria present in the rhizosphere cooperate with the host plant and offer positive effects on its growth [5]. Plant growth-promoting (PGP) elements are delivered by advantageous bacteria residing within or around the roots of plants, such as in the rhizosphere, known as the “plant growth-promoting rhizobacteria” (PGPR), which guard the plants against abiotic and biotic stressed environments, and also improve their physiological capacities [6]. These useful soil microbes, however plentiful in the rhizosphere, are generally under-exploited as bio-inoculants for upgrading crop production, particularly under abiotic stress [7]. Therefore, nowadays, PGPR are getting much attention among agricultural and environmental research as an eco-friendly tool for increasing crop yield, and also as a component of integrated plant nutrient management systems. Rhizobacteria present in the rhizosphere are found to be an efficient alternative for agro-chemicals. The rhizosphere harbors a wide range of microbes, which directly interact with plant roots, and hence rhizosphere is considered a hotspot of bacterial diversity. Plant roots release several organic nutrients and plant effluents, such as sugars, amino acid, and send signals that stimulate microbial growth and production, which is why they nurture 5 to 10 times more fungi and 10 to 50 times more bacteria than ordinary soil [8]. Since root exudates contain the largest amount of carbon source within the soil, the rhizosphere houses a rich microbial community covering a large number of microbial taxa, associated to plant roots and termed as the rhizo-microbiome. Its composition is different from that of the microbial community of the surrounding soil. As a consequence, bacterial population compete for nutrients released in the rhizosphere [9]. Root branching order characterized by primary (stem-attached large), secondary (primary-attached medium), and fine (secondary-attached hair-like) roots have been reported to influence the abundance of microbial communities in the rhizosphere. Bacterial taxa belonging to *Bradyrhizobium*, *Burkholderia, Pseudomonas*, *Sphingomonas*, and *Streptomyces* were found to be abundant in the small root environment, which might be due to the mineral content and exposure with soil [10]. The composition of the rhizo-microbiome changes along with the plant maturation stages and plant genotype [11]. Microorganisms had been reported to promote plant growth within the rhizo-microbiome through direct or indirect mechanisms [12,13].

The plant-microbe interactions around roots are mostly mutualistic. These interactions help in the colonization at the root surface and support the development of plants [14]. Based on their metabolic activity and functional diversity, PGPR has a beneficial effect on the plant’s growth. They help in plant growth promotion by forming symbiotic associations, nitrogen fixation, phosphate solubilization, and production of essential phytohormone such as indole acetic acid (IAA), abscisic acid, cytokinin, etc. However, they also help plants indirectly through resistance/tolerance to biotic stresses and abiotic stress. This is because they exert biocontrol and have an antagonistic effect by various means, such as by the production of antibiotic, lipopeptide, cell wall degrading enzyme, volatile compound, HCN, and siderophore. They reduce abiotic stresses in plants, as they have a role in increasing tolerance against salt, drought, pesticide, temperature, and metal stresses [15].

Endophytic bacteria are plant-associated bacteria that reside in the internal tissues of the plant without harming it [16]. The nature of their mutualistic association depends on their location in the plant tissue, either intercellularly or intracellularly [17]. The integral intracellular colonization by non-cultivable endophytic bacteria in the cytoplasm and cell wall-plasma membrane peri-space in bananas indicated a mutualistic association between the host and the endophytes. Bacteria colonizing the cytoplasmic space release bacterial metabolites directly to the protoplasm of the host, which could influence gene expression and functioning of the host [18]. Endophytes have been isolated from various species of plants and organs and have mostly been recruited from the rhizosphere of the host plants. Endophytes had been observed to enter host plants through roots and colonize intercellular spaces of the roots [19].

Root endophytic bacteria have different functions, adaptations, specialization, and competence [20]. As these root-endophytes inhabit the localized point of entry or spread within the plant, they produce several bioactive metabolites and hydrolytic enzymes to endure in the environment of the host plant. Mechanisms by which root-endophytes survive with the changing environmental conditions could contribute to better growth, health, and development of the host plants [21]. Root-endophytes possess at least one, or some of the mechanisms of the following functions: (i) production of phytohormone, (ii) biological control of phytopathogens, (iii) supply nutrients nitrogen or phosphate for plants [22]. Their ability to reduce stress ethylene by the synthesis of 1-aminocyclopropane-1-carboxylate (ACC) deaminase, production of siderophores, and phosphate solubilization may also contribute to the growth and development of plants [23]. Application of diazotrophic endophytic bacteria could provide nitrogen, as the interior of the plant has direct accessibility of the fixed nitrogen to the plants [24].

Intracellular endophytic bacteria (*Pseudomonas fluorescens* SLB4-P, *Pseudomonas* sp. SLB6, and *Pseudomonas* sp. SY1) isolated from *Phragmites australis* were found to improve seedling development, root and shoot growth, and stimulated the formation of root hair when inoculated on rice, Bermuda grass, and annual bluegrass. *Pseudomonas* sp. strain SY1 was found to reduce *Fusarium oxysporum* infection in all three host plants [25]. Fluorescent-labeled non-pathogenic and non-symbiotic *Escherichia coli* Bl21 and *Saccharomyces cerevisiae* had demonstrated that they move in the root cells of *Arabidopsis thaliana* and tomato plants, which could take up these microorganisms into the root cells [26]. Endophytic bacterial communities associated with three varieties of papaya (Arka Surya, Arka Prabhath, Red Lady) were found to be highly diverse with several common phyla, where bacteria were found to be more abundant in the cytoplasmic matrix. The shoot tips of papaya seedlings harbored a variety of bacteria, which was attributed to their intracellular inhabitation. Activation of originally uncultivable microorganisms to cultivation in papaya stocks showed bacterial growth of *Bacillus* (35%), *Methylobacterium* (15%), and *Pseudomonas* (10%). Functional analysis of metabolically active intracellular endophytes also indicated their roles in different plant processes and pathways [27]. Variety of plant species are associated with the diversity of cultivable bacterial endophytes, with most of the members belonging to soil bacterial genera such as *Azospirillum*, *Bacillus*, *Burkholderia*, *Enterobacter*, and *Pseudomonas* [28]. Microorganisms having PGP and biocontrol potential are used in integrated nutrient and disease management. Moreover, future commercialization and use of bio-inoculants in the agricultural sector as an eco-friendly alternative tool will help to improve crop yield as well as the economy.

## 2. Rhizosphere and Root Endophytes

The term “rhizosphere” was first defined by Lorenz Hiltner in 1904 as the region of soil closely associated with plant roots [29]. The term ‘rhizobacteria’ suggests the gathering of rhizospheric microbes around the plant root surfaces [30]. Bacteria inhabiting the rhizosphere are the source of the formation of endophytic bacterial communities, and in plants, roots have the highest occurrence of endophytic bacteria [31]. Endophytic bacteria are located in intra- and inter-cellular regions or the vascular tissue and colonized aerial parts or roots [32]. Bacteria make entry to plant tissues especially through lateral roots or root hair cells. The entry of endophytic bacterium *Enterobacter asburiae* JM22 in cotton plants was assisted by its ability to hydrolyze plant cell wall-bound cellulose [33]. Root endophytes colonize and penetrate the epidermis of the lateral root below the root hair zone and in root cracks. They have the potential to establish populations both inter-and intracellularly [34]. Interaction of host plants with endophytes is facilitated by bacterial motility, or by the growth of the roots that allow passive colonization. A shift in cellular and molecular levels that occurs during the development of plants [35] may also affect the endophytic colonization. The enzymatic mechanisms have key roles in enabling the endophytic microorganisms to penetrate the plants, which could later be transmitted by seed [31]. The plant root not only provides the mechanical support and nutrients uptake for the plants but also incorporates, accumulates, and emits different types of chemical compounds in form of root exudates [36]. These root exudates act as a chemoattractant for a diverse group of soil microbial communities. The exudation of a diverse group of chemical compounds regulates soil microbial communities by changing soil physical and chemical properties [37]. Additionally, microbial interactions in the rhizosphere influence the rooting pattern and also enhance the supply of accessible supplements to plants [38]. The microbes utilize the root exudates in the rhizosphere as carbon and nitrogen sources. In return, plants take up organic molecules for their growth and development, derived by microbes [39]. The microbial communities might secure up to 15% of the root surface, as they also have the metabolic flexibility to adjust the quality and quantity of the root exudates for their use [40]. The components of exudates can be named allelochemicals, phytotoxins, phytoalexins, phytohormones, and/or ectoenzymes. The measures of exudates vary in the plant to plant, plant growth cycle, and rooting pattern [37].

### 2.1. Root Endophytes as Plant Growth-Promoting Rhizobacteria (PGPR)

Kloepper and Schroth [41] proposed “Plant growth-promoting rhizobacteria (PGPR)” as a group of rhizospheric bacteria that colonizes around the plant roots and enhances plant growth. The PGPR attached to the surface of plant roots is termed as extracellular PGPR. At the same time, PGPR that is localized inside the plant cells and produces nodules are known as intracellular PGPR [42]. Around 2 to 5% of rhizospheric bacteria belong to the group of PGPR [43]. The majority of the PGPR belong to the genera of *Acinetobacter*, *Arthrobacter*, *Azospirillum*, *Azotobacter*, *Bacillus*, *Bradyrhizobium*, *Burkholderia*, *Caulobacter*, *Chromobacterium*, *Enterobacter*, *Erwinia*, *Flavobacterium*, *Frankia*, *Klebsiella*, *Micrococcus*, *Pseudomonas*, *Rhizobium*, and *Serratia* [41].

In the past decade, several studies have been done to understand the functions within the rhizosphere, as it is an ecological niche for microbes that now receives much more attention due to its prospects in the development of sustainable agricultural practices. PGPRs have emerged as sustainable devices for the improvement of farming frameworks. PGPR improves plant growth and development in three ways, i.e., synthesizing specific compounds for the plants, encouraging the take-up of particular supplements from the soil, and decreasing the activity of disease-causing microbes [44]. The PGPR may impact the plant development directly by fixing environmental nitrogen, solubilizing insoluble phosphates, secreting phytohormones, for example, indole acetic acid (IAA), gibberellic acid (GA), and ACC (1-Aminocycloprapane-1-carboxylic acid) deaminase synthesis, which helps in the regulation of ethylene biosynthesis while also indirectly helping siderophore production, induced systemic resistance (ISR), and the production of antifungal metabolites (for example, antibiotics) to suppress the infectious microbes. Rhizospheric bacteria had been reported to enhance growth in several crop plants such as rice, wheat, maize, pea, etc., by colonizing the rhizosphere [45]. Some of the endophytes and their beneficial interaction with the host plants have been summarized in Table 1.

Rhizospheric bacteria and endophytic bacteria may utilize similar mechanisms to promote plant growth promotion. The main difference is that endophytic bacteria are not exposed to uncontrolled changes in soil conditions. Variations in soil pH, temperature, and water content may hinder the proliferation of rhizospheric bacteria and existing soil bacteria may compete for binding sites on the root surfaces of the host plants [7]. Moreover, the use of endophytic bacteria could facilitate the growth of the plants in agriculture, horticulture, silviculture, and also to remove pollutants from the environment, as they are more persistent [59]. PGPR modifies the chemical composition of root cell walls that might allow their progression between root cortex cells and act as endophyte [60]. Such modifications of root cell wall ultrastructure are mainly PGPR generated changes in plant gene expression. Rice roots inoculated with endophytic PGPR *Azospirillum irakense* were observed to increase the expression of polygalacturonase genes [61]. 

### 2.2. Mode of Action of Endophytic PGPR

**Figure 1:**
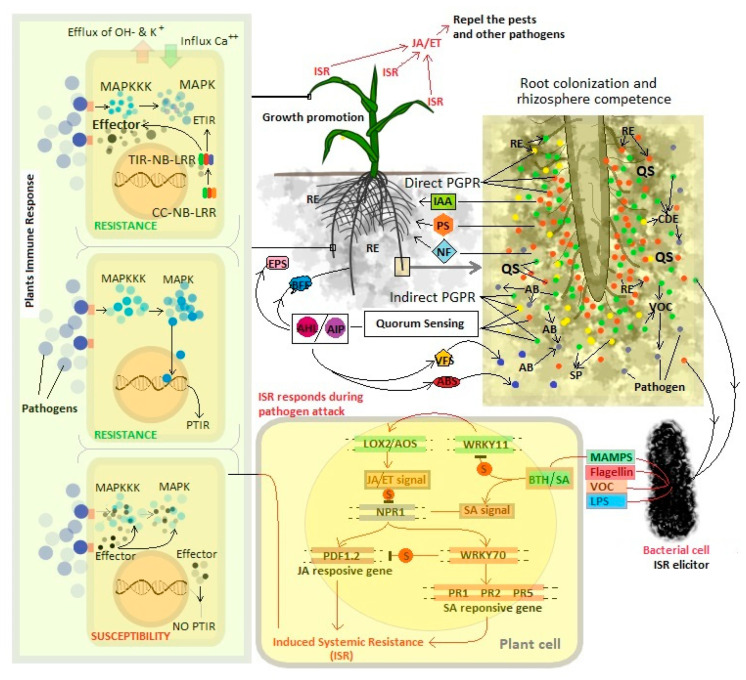
Microbial interactions at the root zone, and cellular responses of the host plant. Abbreviations: JA/ET—Jasmonate/Ethylene, SA—Salicylic acid, AB—Antibiotic, ABS—Antibiosis, RE—Root exudates, VOCs—Volatile organic compounds, VFS—Virulence factor secretion, CDE—Cell wall degrading enzymes, BFF—Biofilm formation, PS—Phosphate solubilization, SP—Siderophore production, NF—Nitrogen Fixation, QS—Quorum sensing, BTH—Benzothiadiazol, LPS—Lipopolysaccharide, IAA—Indole acetic acid, S—Suppression, PTIR—Pathogen Triggered Immune Response, NB—Nucleotide binding, CC—Coiled coil, LRR—Leucine rich repeats.

PGPR can enhance plant growth by employing one or more of these mechanisms (Figure 1).

#### 2.2.1. Direct Mechanism

##### Nitrogen-Fixation

The nitrogen inadequacy in soil vis-a-vis demand for agri-products has prompted the use of enormous amounts of nitrogenous chemical fertilizers to accomplish the targets of yield. Plants assimilate nitrogen mostly in the form of ammonia. A wide variety of nitrogen-fixing bacteria have been recognized, which restore nitrogen symbiotically with specific plants (primarily legumes). Examples of symbiotic nitrogen fixers are *Rhizobium*, *Sinorhizobium*, *Azorhizobium*, *Allorhizobium*, *Mesorhizobium*, *Bradyrhizobium*, *Frankia*, *Azoarcus*, *Achromobacter*, *Burkholderia*, and *Herbaspirillum* [45]. Ammonia production by PGPR is an important feature that indirectly contributes to plant growth. PGPR strains belonging to *Bacillus*, *Pseudomonas*, and *Serratia* had been reported to produce ammonia [62]. Deamination or ammonification processes are carried out by complex nitrogenase enzymes. Nitrogenase (*nif*) genes involved in nitrogen fixation require structural as well as regulatory genes. The structural genes involved in Fe protein activation, biosynthesis of Fe-Mo cofactor, and donation of electrons while regulatory genes regulate the synthesis and function of enzymes [63]. Rhizobia act as endophytes in nodules that promote plant growth and isolation of endophytic rhizobia from nodules of *Vicia* were classified into *Ensifer*, *Shinella*, and *Rhizobium tropici* [64]. Nitrogen-fixing endophytes of the *Rhizobiaceae* family have been observed to have a unique symbiotic relationship with their host plants [65]. Rhizobial endophyte *Azorhizobium caulinodans* ORS571 secreted cellulases and pectinases, which help the bacteria in colonizing the xylem elements of *Sesbania rostrata,* in the process of nodule formation. It indicated that vascular rhizobial endophytes could be symbiotic, and provide fixed nitrogen to their host plants, and that xylem elements are the sites for nitrogen fixation by diazotrophs, as xylem serve as a site for the metabolites exchange, which is essential for nitrogen fixation [57]. Endophytic bacterium *Gluconoacetobacter diazotrophicus* (*Acetobacter diazotrophicus*) could fix nitrogen in sugarcane [66]. Endophytic diazotrophs *Azoarcus* increased nitrogen fixation in kallar grass and increased the productivity of non-legumes including cash crop plants [67]. *Azoarcus* spp. were found to be more abundant in rice roots expressing high levels of nitrogenase in the aerenchyma to indicate the location within the roots [68]. The process of nodulation and N_2_-fixation by endophytic rhizobia has been well studied and there are several excellent reviews available [69,70,71], and it is therefore not elaborated here (Figure 1).

##### Solubilization of Phosphate

Phosphorus (P) is the second most important element, supplemented to soil for plant growth, after nitrogen. This low accessibility of phosphorus to plants is due to its insoluble forms in soil. In contrast, plants assimilate it just in two soluble ways, i.e., monobasic (H_2_PO_4_) and the dibasic (HPO_4_) ions of phosphate [72]. To overcome P insufficiency in soils, there are extensive uses of phosphate fertilizers in farming fields. Plants retain low measures of applied phosphate fertilizers, and the rest are quickly changed over into insoluble forms in the soil. The regular use of phosphate fertilizers is not only expensive but also environmentally undesirable [39]. The microbes, having phosphate-solubilizing activity termed phosphate-solubilizing microorganisms (PSM), may provide the accessible phosphate for the plants [73]. There are different PGPR genera such as *Serratia*, *Pseudomonas*, *Rhizobium*, *Bacillus*, *Azotobacter*, *Burkholderia*, *Enterobacter*, *Bradyrhizobium*, *Streptomyces*, *Cladosporium*, etc., which are the most efficient phosphate-solubilizing PGPR [74]. PGPR employs different mechanisms to solubilize the insoluble phosphates. One of the critical mechanisms is the production of organic acids during sugar metabolism. The rhizosphere inhabiting PGPR exploits the sugars of plant root exudates and releases various organic acids. It creates an acidic condition by lowering the pH, and then the organic acids act as chelating agents and release phosphates from insoluble phosphate compounds. The synthesis of phosphatases by PGPR also mineralizes phosphates from the organic phosphatic substances [39].

Endophytes had been known to increase the availability of phosphorus to the plant through phosphorus solubilization. The release of low molecular weight acids enables the chelation of metal cation attached to phosphorus and creating easily accessible to plants [75]. Endophytic isolates *Achromobacter xiloxidans* and *Bacillus pumilus* were characterized for phosphate solubilizing abilities in sunflower [76]. Endophytic bacteria isolated from soybean can assimilate phosphate [77]. Oteino et al. [78] described the genetic systems of endophytic strains that can solubilize phosphate and observed that *Pisum sativum* L. plants treated with endophytic strains showed a significant increase in plant growth parameters. Five *Pseudomonas* strains were able to solubilize tricalcium phosphate [Ca_3_(PO_4_)_2_] to the level of >400 mg·L^−1^ while *Bacillus* showed poor solubilization in their study. Three *Pseudomonas* strains (L111, L288, and L321) were observed to possess *pqq* gene clusters (*pqqFABCDE*) and *gcd* and *gad* genes for phosphate solubilization. The presence of *pqq* genes in association with the *gcd* gene confirmed that the strains could produce gluconic acid, which is the main mechanism for phosphate solubilization.

##### IAA Production

IAA (a type of auxin) is known for its role during the various developmental stages of plants such as cell division, cell elongation, and tissue differentiation. IAA is produced by multiple strains of bacteria that are known for their plant growth-promoting activity [79]. PGPR strains help plants to uptake nutrients, increase root size, length, and biomass as they have a close association with root and root-soil [80,81]. Endophytic bacteria including *Bacillus aryabhattai*, *B. subtilis*, *Klebsiella pneumoniae*, *Microbacterium trichotecenolyticum*, and *Paenibacillus kribbensis* were reported to produce IAA [82]. Tryptophan is the natural exudate from the roots of the plants, and it is the main precursor for the biosynthesis of IAA. Several pathways using tryptophan as a precursor for IAA syntheses such as indole-3-acetamide (IAM), indole-3-pyruvate (IPyA), tryptamine (TAM), tryptophan side-chain oxidase (TSO), and tryptophan independent pathways have been reported [79]. The PGPR, having the IAA production ability, might have multiple roles in different kinds of plant-bacteria communications, during plant development, and root nodulation [83]. The amount of IAA produced by different endophytic bacteria and plants affect their interaction [84] (Figure 1). Microbial regulation on the IAA signaling system has been considered as the key mechanism that enhances lateral root development and relieves plant stress [7]. Endophytes that excessively produce IAA have exhibited transcriptional variations in nitrogen-fixing nodules [85]. They could increase the expression of the *nifH* gene, which leads to higher nitrogenase activity in rice plants [86]. IAA producing the traits of endophytic bacteria could increase the cadmium metal uptake in the root cell wall and alleviated metal toxicity by translocation from root to shoot in Arabidopsis [87]. Endophytic bacteria identified as *B. aryabhattai* MBN3, *B. megaterium* MJHN1, and *B. cereus* MJHN10 were found to produce IAA through a tryptophan dependent pathway. In-vitro application of the isolates in the plant roots was observed to increase the numbers of lateral roots and enhance root length [88]. Endophytic bacteria isolated from terrestrial orchids were observed to produce IAA and stimulated root development and root length in kidney beans [89]. Besides, bacterial IAA expands root surface territory and length, allowing the plant to take-up better supplements and improve plant growth [90]. It was also considered that IAA production is an effective tool for the detection of beneficial microbes with the potential for plant growth promotion [91]. Plant growth-promoting endophyte *Bacillus* sp. SLS18 produced IAA, siderophores, and ACC deaminase and increased the biomass of sweet sorghum (*Sorghum bicolor* L.), *Phytolacca acinosa* Roxb. and *Solanum nigrum* L. in presence of Mn/Cd, as the strains showed resistance to heavy metals [92]. Seeds of *Azadirachta indica* A. Juss (Meliaceae) treated with three endophytic *Streptomyces* strains AzR-051, AzR-049 and AzR-0109 were observed to promote growth and antagonized the growth of *Alternaria alternate*, which is the causal agent of early blight disease of tomato plants. The strains AzR-051, AzR-049, and AzR-0109 were found to produce IAA at 13.73 µmol mL^−1^, 9.22 µmol mL^−1^, and 10.43 µmol mL^−1^ respectively [93]. The cultivable rice seeds endophytes exhibited promising plant growth-promoting activities, which were observed to be dominated by Proteobacteria of the class Gamma-proteobacteria [94]. Plant growth-promoting endophytic bacterium, *Sphingomonas* sp. LK11 isolated from *Tephrosia apollinea* leaves produced IAA (11.23 ± 0.93 μM/mL) and exhibited physiological active gibberellins (GA_4_: 2.97 ± 0.11 ng/mL) and inactive gibberellins (GA_9_: 0.98 ± 0.15 ng/mL; GA_20_: 2.41 ± 0.23 ng/mL) [95]. Type strains belonging to *Flavobacterium* sp. were found to be IAA producers and phosphate solubilizers with tolerance to high salt concentration and osmotic stress [94].

##### 1-Aminocyclopropane-1-carboxylate (ACC) Deaminase

Ethylene is a vital plant phytohormone that facilitates plant growth and development under non-stressed conditions. However, under stressed conditions, the level of ethylene increases and negatively regulates plant growth. It restricts the elongation of root and transport of auxin, supports aging and extirpation of organs, and assists in the ripening of fruits. Ethylene plays a key role in the activation of plant defense against biotic stresses [96]. 1-Aminocyclopropane-1-carboxylate (ACC) is the primary precursor in plants for ethylene synthesized by ACC synthase by converting S-adenosyl methionine. Under stressed conditions, the activity of ACC synthase increases and produces a high amount of ethylene [81]. When the ACC is degraded by the ACC deaminase producing bacteria, the level of ethylene decreased, which results in the elongation of roots [7,97]. A diverse group of PGPR having ACC deaminase activity has been reported as *Serratia*, *Pseudomonas*, *Bacillus*, *Acinetobacter*, *Rhizobium*, etc. [39]. This PGPR with ACC deaminase activity hydrolyses the primary precursor ACC to α-ketobutyrate and ammonia and reduces the ethylene level and improves plant health under stressed conditions [98]. ACC deaminase is a stress release enzyme, as it alleviates different types of biotic and abiotic stressors such as pathogenic attacks, drought, metal, radiation, salt, heat stress, etc. [6]. 

Endophytic microorganisms with the ability to utilize ACC as the nitrogen source could reduce the level of ACC and ethylene that led to the prevention of ethylene mediated plant growth inhibition. Physiological and molecular characterization of endophytic bacteria such as *Enterobacter*, *Klebsiella*, and *Pseudomonas,* with the ability to produce ACC deaminase, have been reported [99,100], and genes responsible for its production have been isolated and expressed from *Klebsiella* sp. [101]. Treatment with *Azospirillum brasilense* Sp245 expands the ABA content in *Arabidopsis thaliana* [102]. A study conducted by Rashid et al. [103] isolated a total of 174 bacterial endophytes from internal tissues of tomato plants, and 25 strains were observed to possess ACC deaminase activity with intracellular levels ranging from 0.43 to 12.50 µmol^−1^g^−1^h. The strains identified as *Pseudomonas* spp. showed higher ACC deaminase activity while *Microbacterium* spp., *Bacillus* spp., *Agrobacterium* spp., exhibited higher levels of IAA and siderophore.

##### Role of Bacteria in Root System Architecture (RSA), and Its Modification

To assist growth, different types of plants enhance their root arrangement to transverse soil and obtain nutrients. The root is an integral part of plants comprising distinct regions, for instance, the tip of the root, meristem of the root, elongation region, and emerging oblique roots [104]. Different regions of the root perform differently. The root hairs are epidermal cells essential for mineral nutrition in a plant [105,106]. In Fabaceae, the root tip is the crucial section to initiate the process of colonization of rhizobia, which ultimately triggers root nodule emergence [107]. In the case of Poaceae, PGPR colonizes the oblique roots and root hairs where they illustrate the resources that are plant favorable [108]. RSA includes topography of root arrangement, primary and oblique roots distribution, and the amount and realm of several roots. RSA holds an impact on the various biotic and abiotic agents, involving plant growth-promoting bacterial strains. PGPR, along with their interference with plant hormonal balance, remodel RSA, and root tissue structure. PGPR modifies the chemical configuration and the structure of the cell wall of the root. For instance, *Bacillus pumilus* INR-7, which is a biocontrol agent, can magnify the deposition of lignin in epidermal tissues of pearl millet [109]. *B. pumilus* SE34 and *B. subtilis* UMAF6639 triggered a similar reaction when injected into the roots of pea and melon. After analysis, it was concluded that the root injected with *Azospirillum lipoferum* CRT1 had high lignin content compared to the uninjected ones. In the root cell wall, pectate is lowered by pectate lyases, which is synthesized by *Azospirillum irakense*. It shows promotion between the cortexes of roots and behaves as an endophyte [60]. *B. subtilis* GB03 encourages the growth of *Arabidopsis* growth by forming VOCs, which regulates the expression of 38 genes [110]. Out of them, 30 were involved in the extension of the cell wall. The expression of polygalacturonase genes injected in rice roots is stimulated by the *A. irakense* [61]. 

RSA is also affected when PGPR, having the ability to produce plant-hormones, invade or colonize root tissues [111]. For the plant organogenesis and root shape architecture, the equilibration between phytohormones such as auxin and cytokinin is an essential regulator [112]. Endophytic bacteria affect the auxin to cytokinin ratio because they create a broad span of plant hormone and induce phytohormones and subsidiary metabolite, which inhibits the pathway of plant auxins such as 2,4-diacetylphloroglucinol and nitric oxide. The auxin formed by many plant-associated bacteria is indole-3-acetic acid [79]. In the growth and development of a plant, extrinsic IAA controls a diverse variety of processes such as primary root elongation, which is supported by the low concentration of IAA, whereas the formation of oblique roots decreases the length of the root and expands root hair establishment, which is stimulated by the high concentration of IAA. *Azospirillum brasilense* can reduce nitrate, which ultimately generates NO during the colonization of roots [109]. Lateral root formation is controlled by NO, which is intricate in the pathway of auxin signaling. DAPG acts as an indicating molecule for systematic resistance incited plants [113], inciting exudation of roots [114], and strengthening the branching of roots. The inoculation of bacteria may have an effect on auxin signaling pathways and lateral root response of the host plant. In *Arabidopsis*, inoculation of *Phyllobacterium brassicacearum* STM196, a low-IAA producer, triggered changes in IAA distribution in plant tissues, which was independent to IAA released by bacteria itself [115]. Cytokinin production (especially zeatin) has been observed in root-associated bacteria such as *Arthrobacter*, *Pseudomonas fluorescens*, etc. [116,117]. It supports plant cell division, and also influences the multiplication of root hairs, and controls the differentiation of root meristem. 

#### 2.2.2. Indirect Mechanisms

##### Siderophore Production 

Iron is one of the most abundant minerals found on the earth’s surface. It is classified as a micronutrient yet is not easily available for plants. Iron is usually present in nature as Fe^3+^, which is exceptionally insoluble; to take care of this issue, PGPR releases siderophores [118]. Siderophores are low atomic weight compounds that chelate ferric iron (Fe^3+^). Microbial siderophores facilitate the Fe supply to plants in iron deficit conditions and improve plant growth [119] (Figure 1). Under Fe^2+^ limited conditions, microbial siderophores form complexes with Fe containing minerals or organic compounds, which is then taken up by microbial cells where Fe^3+^ is converted and released as Fe^2+^ [120]. Siderophore- producing PGPR restricts the growth of several plant pathogens, by creating a competitive, iron-limiting environment [121]. The metal stress condition induces microbial siderophore production in the rhizosphere. It has been observed that under a heavy metal stressed condition, plants experience iron deficiency and microbial siderophore helps the plants to maintain sufficient Fe^2+^ condition [39]. Bacterial siderophore production may be influenced by the presence of heavy metals, and plants could reduce heavy metal-toxicity by improving iron supply to the plants [122]. In addition to iron, siderophore could form stable complexes with heavy metals, which lead to an increase in the soluble metal concentration and thus bacterial siderophores assist in alleviating plant stress [123]. Endophytic bacteria such as *Methylobacterium mesophilicum* and *Sphimgomonas* spp. were found to be resistant to nickel (Ni), as their endophytic lifestyle ensure survival in low levels of free iron content in the plant tissue [124]. Siderophore producing Lead (Pb)- resistant endophytic *Pseudomonas fluorescens* G10 and *Microbacterium* sp. G16 inoculated in *Brassica napus* were found to increase Pb uptake into the shoots and enhanced Pb availability in *B. napus* [125].

*Pseudomonas fluorescens* spp. is an efficient iron (Fe^3+^) competitor that produces two types of siderophore-pyoverdine (fluorescent pigment) and pyochelin (non-fluorescent pigments) [126]. Endophytic actinomycetes can promote plant growth and have fungicidal properties for *Pythium* diseases in cucumber [127] and wheat [128]. Further, *Pseudomonas putida* B10 help by suppressing the growth of *Fusarium oxysporum* by siderophore production in the soil [129]. In addition, endophytic bacteria that produce siderophore could restrict the growth of plant pathogens as they create an iron-limiting microenvironment [130]. Jasim et al. [100] studied the endophytic bacterial isolates of ginger, and the isolates ZoB1 (*Bacillus* sp.), ZoB2 (*Pseudomonas* sp.), and ZoB3 (*Stenotrophomonas* sp.) that produced siderophore. *Bacillus* sp. had been reported to produce petrobactin type of siderophore [119]. *Pseudomonas* sp. had been described to produce more than 50 structurally related siderophores including pyoverdins [131]. Genome sequencing of *Strenotrophomonas maltophilia* have shown its capability to produce catechol type of siderophore compound enterobactin [132]. Siderophores were observed to be efficiently produced by rice seed’s endophytic *Pseudomonas* strains [94].

##### HCN Production

HCN producing PGPR act as biocontrol agents by showing harmful effects against the plant-parasitic microbes [95]. The benefits of HCN production in bacteria itself are not clear to date, but the role of HCN against the fungal attack in plants has been reported in several PGPR strains [133]. Rhizospheric plant beneficial microbes with HCN production ability have a significant role in plant protection against several plant diseases. Certain endophytes such as *Bacillus* produce HCN in avocado and black grapes [134] and HCN produced by *Pseudomonas putida* revealed antibacterial activity against *Escherichia coli* and *Klebsiella pneumoniae* and antifungal activities against *Pythium* ultimum [134]. Endophytic bacteria identified as *Pseudomonas* and *Serratia*, isolated from within the tissues of various plants revealed improvement in seed germination, seedling length, and plant growth of oilseed rape and tomato. Seeds treated with endophytes were observed to reduce disease symptoms caused by vascular wilt pathogens *Verticillium dahlia* Kleb and *Fusarium oxysporum* f. sp. *lycopersici* (Sacc.). Endophytic isolates were observed to produce microbial inhibitory volatile compounds and HCN that could suppress plant pathogens. In addition, endophytes residing in the vascular tissue (conductive tissue) produce induced resistance, which enables them to inhibit soil-borne pathogens that are colonized in the vascular tissue of a plant [135].

##### Cell Wall Degrading Enzymes

It has been contemplated that numerous PGPR integrate extracellular hydrolytic enzymes that are engaged with the degradation of fungal cellular components, for example, chitin, hemicellulose, cellulose, proteins, and DNA [136]. Secretion of hydrolytic enzymes by endophytes appears to be important for colonizing the plant roots [137]. The capability of the endophytes to release lytic enzymes facilitates the hydrolysis of a wide range of polymeric compounds including chitin, proteins, cellulose, hemicellulose, and DNA [138]. When endophytes colonize on the plant surface, they produce enzymes that could hydrolyze the plant cell wall. Further, the hydrolytic enzymes produced by endophytes have the capability of repressing plant pathogens directly by degrading cell walls of fungi and oomycetes [139]. Endophytic bacterial colonization in the plant tissues occurs through migration along with intercellular spaces by secreting cell-wall degrading enzymes such as cellulases and pectinases [20]. The cell wall of fungi (except for oomycetes) is composed of chitin [139]. The capability of hydrolytic enzymes produced by PGPR to break down the glycosidic bonds of chitin gives it immense importance as a biocontrol agent [140]. The lytic activity of the hydrolytic enzymes on the fungal cell wall, hyphal tip, and germ tube cause hyphal swelling, twisting, and bursting of the hyphal tip leading to fungal death. Among the immense number of hydrolytic catalysts, chitinase, protease, glucanase, and cellulase are significant because of their capacity to degrade and lyse the cell wall of fungal parasites [138]. Cell degrading enzymes of rhizobacteria harm the basic structure of the cell wall of phytopathogens (Figure 1). Various PGPR strains (including endophytes) belonging to the genera of *Serratia*, *Pseudomonas*, *Rhizobium*, *Bacillus*, *Azotobacter*, *Burkholderia*, *Enterobacter*, *Bradyrhizobium*, *Streptomyces*, *Cladosporium*, etc., can secrete hydrolytic enzymes for the biocontrol of plant fungal pathogens such as *F. oxysporum*, *R. solani*, *P. ultimum*, *S. rolfsii*, etc. [141,142]. Plant-beneficial endophytic bacteria including members of *Enterobacter*, *Pseudomonas*, *Burkholderia*, *Bacillus*, and *Azospirillium* have been identified from various plant species that act as biocontrol agents [143].

Hydrolytic enzymes (chitinase, protease, glucanase, and cellulase) secreted by PGPR are responsible for the lysis of fungal pathogens during hyperparasitism [144]. Some of the PGPRs, such as *Serratia* sp. [145], *Pseudomonas* [146], *Paenibacillus* sp. [147] are also biocontrol agents (BCAs), which release hydrolytic enzymes, and are utilized in the biocontrol of phytopathogens [148]. Endophytic PGPR *Streptomyces anulatus* S37 isolated from wild *Vitis vinifera* was found to confer resistance against *Botrytis* *cinerea* pathogen [149]. Mutational inactivation of 1,3-glucanase gene in *Lysobacter enzymogenes* showed a reduction in biocontrol activity toward *Pythium* damping-off disease in sugar beet and bipolaris leaf spot of tall fescue [150]. Lytic enzymes produced by endophytic *Streptomyces* revealed a strong effect on antagonizing cacao witches broom disease [151], found to affect inter/intracellular colonization [152]. 

##### Antibiotic Production

The production of a diverse group of antibiotics is one of the significant PGP characteristics, restricting plant parasites, particularly fungi [153]. The use of PGPR for controlling infection in agricultural plants has been proposed as an alternative way for chemical pesticides. Pyrolnitrin secreted by *Pseudomonas* species and *Burkholderia* exhibited antagonism against a wide range of fungi belonging to the group of Ascomycetes, Basidiomycetes, and Deuteromycetes, including some of the phytopathogens such as *Botrytis cinerea*, *Rhizoctonia solani*, *Sclerotinia sclerotiorum*, and *Verticillium dahliae* [154]. Similarly, 2,4-diacetylphloroglucinol (2,4-DAPG), a polyketide antibiotic produced by several microbes, shows antibacterial, antifungal, antihelminthic, antiviral, as well as phytotoxic effects [155]. Some endophytic strains of *Pseudomonas* have shown their ability to synthesize chemicals such as phenazine that enhance the growth of the host plant [156]. Phenazines (phenazine-1-carboxylic acid, PCA; 2-hydroxyphenazine-1-carboxylic acid; and 2-hydroxyphenazine), a diverse group of antibiotics, show antagonisms against a diverse group of phytopathogenic fungi, gram-positive, as well as gram-negative bacterial pathogens [157]. PGPR belonging to the genera of *Bacillus* also produce a diverse group of antimicrobial metabolites such as subtilisin A, subtilin, TasA, and sublancin, which are derived from the ribosome or the non-ribosomal peptide and/or polyketide synthetases (NRPSs/PKS) Several antibiotic coding genes have been identified in *Bacillus amyloquefaciens* FZB42, and *Bacillus subtilis* 168, which are *act*, *bac*, *bae*, *bmy*, *dfn*, *dhb*, *fen*, *mln*, and *srf,* biosynthesized by the NRPSs and PKS enzymes [36].

Endophytes produce natural products such as alkaloids, flavonoids, terpenoids, steroids—natural products that are antibiotics, biological control agents, anticancer agents, and other bioactive compounds [158]. Endophytic bacteria produce signals that could mediate crosstalk with the host and lead to develop resistance against infections [159] (Figure 1). Endophytic *Streptomyces* sp. isolated from vine *Monstera* sp. produced a complex novel peptide antibiotic with activity against pythiaceous fungi and *Cryptococcus neoformans,* which is a human fungal pathogen [160]. Endophytic *Bacillus* sp. have been described to possess antifungal activities against plant pathogens. Endophytes isolated from older balloon flower plants antagonize *Phytophthora capsii*, *Fusarium oxysporum*, and *Pythium ultimum* [161].

##### Volatile Organic Compounds Production

Different types of volatile compounds (VOCs) are released by the plant-associated bacteria and their main function is to act as signal molecules and help in cellular-communications [162]. However, these VOCs have also been reported to restrict the growth of plant pathogens [163]. Different bacterial species, for example, *Bacillus*, *Pseudomonas*, *Erwinia*, *Staphylococcus*, etc., have been identified to produce 346 types of distinct volatile compounds [164]. *Bacillus* endophytes exhibited biocontrol activity by producing VOCs, which could protect plants directly against phytopathogens or indirectly by inducing plant resistance [165,166] (Figure 1). VOCs emitted by *Bacillus* sp. were reported to modify root architecture, stimulate fresh weight, primary root length, and lateral root number and length on *A. thaliana* [167]. 2,3-butanediol produced by endophyte *Enterobacter aerogenes* was found to influence resistance to pathogens and herbivorous insects and disturbs tritrophic interactions [168]. It is found that after 96 h of disease induction in plants, the VOC’s namely acetoin and 2, 3-butanediol reduce the growth pathogens under growth-chamber conditions [169]. Endophytic PGPR *Bacillus subtilis* strain DZSY21 was reported to inhibit the mycelia growth and conidial sporulation of fungal pathogen *Curvularia lunata* by producing VOCs like 2-methylbutyric acid, 2-heptanone, and isopentyl acetate. Application of VOCs in maize leaves was observed to reduce disease indexes from 60.52 to 26.64%, further isopentyl acetate showed enhancement in accumulation of intracellular reactive oxygen species (ROS) in conidia [170]. VOCs (2,5-dimethylpyrazine and benzothiazole) produced by endophytic PGPR *B. velezensis* ZSY-1 was found to have strong antifungal activity toward *Alternaria solani* and *Botrytis* *cinerea* [171]. Moreover, fungal endophyte *Muscodor albus* produced VOCs including 2-butanone and 2-methyl furan, which have antibiotic properties [172]. Although more research is needed for further understanding of the volatile compounds, it is clear that VOCs work to enhance the plant’s self-immunity.

## 3. Role of Microbial Signals Modulate PGPR Functions

### 3.1. Regulation of Quorum Sensing by Plant-Associated Bacteria

A population density-based phenomenon known as quorum sensing (QS) makes bacteria able to communicate by synthesizing small signaling molecules. Most widely studied are acyl-homoserine lactone (AHL) molecules produced by Proteobacteria, which move in and out of the cell either passively or actively. Signaling compounds involved in the QS process are called autoinducers [173]. In this process, Gram −ve bacteria use acyl-homoserine lactone (AHL), and Gram +ve bacteria use auto-inducing peptide (AIP) molecule as signaling molecules. The QS Process depends upon the cell density of bacteria. Plant growth and development are also improved by QS. PGPR colonization in the rhizosphere of the plant root exudates is also mentored by Quorum sensing [174]. In the colonization and biofilm formation process on the root surface, plant-associated bacteria used AHL biosensors to produce the AHL substances [175]. QS regulates the process of antibiosis, biofilm formation, exopolysaccharide production, virulence factors secretion, bioluminescence, sporulation, and competence process, in addition to the expression of many PGP attributes. PGPR may influence the cell-to-cell communications among itself and other bacteria and fungi, inhabiting and sharing the microenvironment of roots. This is facilitated through changes in the bacterial density by synchronizing gene expression [176], while some of the bacterial signals are not related to cell density, they may still use chemical signals such as AHL for this purpose. AHL production is commonly found in endophytic *Pseudomonas* spp. than soil-borne *Pseudomonas* spp. and such strains are more available in plant tissues than in the rhizosphere [177]. It has been reported that signaling compounds secreted by one species could induce density-dependent responses in other species [178]. In *S. plymuthica*, an endophyte of rice plants, QS was observed to regulate the antifungal activities by affecting the exoenzymes release, though it was unfavorable towards the production of IAA [179]. *Azospirillum lipoferum* B518, an endophyte of rice plants was able to release AHL signals [180] and thus terminate the pectinase activity, in addition to enhanced synthesis of siderophore and reduction in the production of IAA. However, it has no impact on cellulase activity [181]. Crosstalk among species having a similar AHL signal or framework has been noticed within the root-associated bacteria of wheat and tomato [182]. AHL-mimics impede bacterial QS and regulate the assertion of plant-beneficial activities [183]. 

### 3.2. Role of Quorum Sensing in Plant Defense and Biocontrol

Cellular signal molecules play a key role in regulating plant immune responses and reduce infection by retarding the pathogen proliferation. Bacterial cyclopeptide (CDP), N-acyl-L-homoserine-lactone, AHL produced by QS helps the plant to induce its defense responses. In the case of the ISR of plants, the QS response of bacteria is thought to be of great importance. Endophytic bacteria have been reported to initiate ISR mediating different pathways salicylic acid (SA), Jasmonic acid (JA), and ethylene (ET), which are the signaling pathways involved in ISR induction [184] (Figure 1). Different members of the root endophyte, for example, *Pseudomonas, Serratia, Burkholderia* [185,186,187] have been found effective in inducing plant defense with QS response. The role of QS in ISR elicited by *Serratia marcescens* strain 90–166 in two tobacco plants harboring genes for either AHL degradation (AiiA) or AHL production (AHL) was examined. Root treated with *S. marcescens* strain 90–166 showed increased ISR to the bacterial pathogens (*Pectobacterium carotovorum* subsp. *carotovorum,* and *Pseudomonas syringae* pv. *tabaci*) in AHL plants and ISR was found to be reduced in AiiA plants. On the other hand, bacterial treatment in AHL plants decreased ISR in the *Cucumber mosaic virus*, however; it was improved in AiiA plants [188]. 

The first line of evidence on defense response in plants induced by AHL was demonstrated with AHL producing isolate—*Serratia liquefaciens* MG1, which could suppress the pathogenicity of *Alternaria alternate*, the tomato fungal leaf pathogen, more effectively, than compared to the AHL negative mutant [189]. Endophytic *Serratia* sp. G3 was reported to confer biocontrol activities through AHL-mediated QS molecules. It was found to produce 10 AHLs signal molecules of which the most abundant AHLs detected were 3-oxo-C6-HSL (N-hexonoyl-homoserine lactone), C4-HSL (N-butanoyl-homoserine lactone), C6-HSL (N-hexanoyl-homoserine lactone), 3-OH-C6-HSL (N-3-hydroxy-hexanoyl-homoserine lactone), and 3-oxo-C7-HSL (N-3-oxo-heptanoyl-homoserine lactone) [190]. This led to a new phenomenon of AHL-induced resistance. Another remarkable finding showed the rhizobacteria *Bacillus pumilus* SE3 could cause changes in root cell walls of the plants challenged with *Fusarium oxysporum*, due to increased callose deposition and phenolic compounds, thereby creating hindrance in fungal infection [191]. A similar study was conducted on *Arabidopsis thaliana*. The plant treated with exogenous AHL molecules generated a priming response by enhanced deposition of callose, lignin, and phenolic materials upon bacterial infection [192]. 

## 4. Root Colonization and Rhizosphere Competence

Endophytic colonization involves complex communication between the microbe and the host plant, and usually, it starts by colonizing roots where endophytic microbes require recognition of specific compounds released by the roots [193]. Endophytic bacteria are specialized bacteria that can invade plant roots, and inside the roots they infect adjacent plant tissues [20]. Rhizosphere colonization by PGPR improves plant growth by colonizing the root system and suppresses deleterious rhizosphere microorganisms [194]. PGPR improves the anatomy and plant tissue function present within a particular length from the settled site similar to a shoot; firstly, there is PGPR, which intensifies the intake of nutrients uptake considering the roots of the plant. Alternatively, endophytic plant growth-promoting bacteria trigger plant defense response pathways regulating endophytic colonization. Endophytic colonization by *Klebsiella pneumoniae* 342 activated the ethylene signaling pathway in *Medicago truncatula*. An ethylene insensitive mutant of *Medicago truncatula* was observed to be hypercolonized by Kp342, however, colonization was further found to be reduced with the addition of 1-methylcyclopropene, which is an ethylene function inhibitor. Colonization of *Salmonella enterica* serovar *typhimurium* strain 14,028 (that does not harm plant) was observed to be affected by both salicylic acid (SA)-dependent and independent responses. Mutants lacking extracellular components such as flagella or type III secretion system encoded by *Salmonella* pathogenicity island 1 (TTSS-SPI1) also influenced the endophytic colonization in *Medicago* spp. in either SA-dependent or SA independent responses [195]. Diazotrophic endophytes *Gluconacetobacter diazotrophicus* PAL5 and *Herbaspirillum rubrisulbalbicans* HCC103 inoculation in sugarcane exhibited modulation in the expression pattern of a putative ethylene receptor (SCER1) and two putative ERF transcription factors (SCERF1 and SCERF2). The gene expression profile of these factors could establish efficient or inefficient associations with the diazotrophic endophytes, which shoqed a high or low rate of nitrogen fixation, respectively. This revealed SCER1, SCERF1, and SCERF2 contribution in ethylene signaling cascade(s) that could identify endophytic association [196]. 

Endophytic bacterial colonization in the rhizosphere and their entrance into the endorhiza could progress with the secretion of cell wall degrading enzymes. An endophytic bacterium *B. phytofirmans* strain PsJN, earlier known as *Pseudomonas* sp. [197], was later classified as *B. phytofirmans* PsJN^T^ [198]. The *B. phytofirmans* strain PsJN was observed to enter into the endorhiza by secreting endoglucanase and endopolygalacturonase, endo-β-D-cellobiosidase, and exo-β-1,4-glucanase. Colonization of peripheral cylinders, mainly xylem vessels and endodermis barrier by the endophytic bacteria, allowed it to spread inside plants [199]. Root endophytes were described to colonize and penetrate the epidermis below the root hair zone and in root cracks [34]. The transport of endophytes from seeds into plant tissues and roots had been demonstrated by endophytes labeled with a green fluorescent protein and their movement was observed to continue throughout the root [200]. The *B. phytofirmans* strain PsJN migrated from endorhiza to inflorescence organs of grapevine, which would use non-functional vessels, and the strain was detected in the lumen of xylem vessels, which allowed for bacterial progression within plants [199]. Previously it had been assumed that pathogens move through the xylem vessel and endophytes colonized non-functional vessels or transport through the apoplast to reach the aerial parts of the plant [201].

Diazotrophic endophytic bacteria colonize and modify the environment of the host plant through nitrogen fixation. Transcriptomic analysis has indicated the upregulation of *nif* genes that are involved in nitrogen fixation when bacteria attach to the root surface [202]. Bacteria moving from plant rhizosphere to the endosphere should overcome plant defense responses most importantly through the production of reactive oxygen species (ROS) and reactive nitrogen species (RNS). Endophytic bacteria require detoxification of ROS and RNS to adapt to the environment. The importance of ROS detoxification was observed in *Gluconacetobacter diazotrophicus* PAL5 during root colonization by superoxide dismutase and glutathione reductase [203].

## 5. Endophytic Arbascular Mycorhiza (AMF)

Arbuscular mycorrhizal fungi and root endophytic fungi are the root symbionts that positively affect plant growth and nutrition [204]. The role of AMF in high phosphorus uptake through activating a specific group of phosphate transporters in plants is well documented [204]. The symbiosis between nitrogen-fixing bacteria (rhizobia and actinorhizas), AMF, and plants affects several functional elements, which includes plant symbiotic signaling pathways, root colonization strategies, the formation of the host-microbe interface, and phytohormone release for root development [205]. Co-inoculation of AMF, *Cochliobolus sativus*, *Diaporthe* sp., and *Phoma exigua* var. *exigua* exhibited a beneficial effect on biomass yield in *Verbascum lychnitis*. AMF was found to increase the rate of photosynthesis and abundance of photosystem II core protein (PsbC) revealed an upregulation in plants when the plants were colonized by *Epichloe typhina,* showing an increase when the negative effect of fungal endophyte was attenuated by AMF [206]. Synergistic interaction of endophytic *B. subtilis* and AMF showed a significant increase in shoot and root dry weight, nodulation, nutrient acquisition and alleviate the adverse effect of saline stress in *Acacia gerrardii* [207]. Bacterial diversity and its effect on mycorrhizal symbiosis has been investigated by Deveau et al. [208]. They suggested that bacterial communities in the bulk soil, sporocarps and ectomycorrhizal (EM) root tips of *Tuber melanosporum* exhibited significant changes in sporocarp formation, while little variation was observed in EMs. AMF represent an important niche for interaction with bacteria because the fungi have a large surface area that allows access to photosynthetically derived carbon to the colonizing endophytic bacteria, as observed in *Pinus sylvestris* [209] and *P. muricata* [210]. High throughput sequencing elucidated the impact of AMF inoculation on indigenous root microbial communities, which showed inoculation modified the abundance of indigenous AMF and other members of fungi and showed enrichment in several bacterial communities with the introduction of new bacterial species. Members of *Microbacterium*, *Cellulomonas*, *Burkholderia*, *Streptomyces,* and *Sphingomonas* were observed to have closely interacted with the introduced AMF while members of *Acetobacteraceae*, *Alicyclobacillaceae, Armatimonadaceae*, and *Methylobacteriaceae* were observed to be reduced with the inoculation [211]. An association of endobacterium *Candidatus* Glomeribacter gigasporarum with AMF *Gigaspora margarita* showed a significant increase in fungal primary metabolism and respiration by 50% [212]. Therefore, the interaction between AMF, endophyte and host plant require attention for its holistic role in the alleviation of stress and plant growth. 

## 6. Endophytic PGPR and “Omics” Technologies

### 6.1. Effect of Root-Metabolome on Root-Microbiome

The microbial populations generate exometabolites, which affect plant growth and other properties. The inoculation of PGP bacteria, including endophytes, has been reported to influence the metabolomic changes in the host plant, which has been described by analyzing the metabolites of root discharge, tissues of root, and shoots. The root enzyme activities including metabolites productivity, mainly flavonoids, can be switched by the action of plant growth-promoting bacteria instigating changes in root discharge pattern [213]. *Chryseobacterium balustinum* Aur9 was found to regulate flavonoid exudation in soybean roots [214], whereas *Chryseobacterium* [214] or *Azospirillum* [215] influenced the discharge of flavonoids in *Fabaceae* roots. *Herbaspirillum seropedicae* inoculated rice plants exhibit elevated contents of malate and amino acids in the shoots of the host plant [216]. Bacterial inoculation also influenced the synthesis of various alkaloids and terpenoid compounds [217]. Similarly, when two cultivars of rice were injected with two different strains of *Azospirillum*, then the metabolic profiles suggested the changes in secondary metabolites, mainly the phenolic compounds [218].

Endophytic fungi *Epichloe typhina* colonization on plant host *Dactylis glomerata* endured improvement in photosynthesis efficiency. The abundance of photosystem II proteins LHCI, LHCII, chlorophyll b, and carbon assimilation were revealed to be increased with endophyte colonization. Its colonization increased the malate export out of the chloroplast where assimilated carbon could be transported through malate from chloroplast to apoplast, where endophyte resides, allowing it to sustain better growth of the host plant [219]. Arbuscular mycorrhizal fungi (AMF) enhanced amino acid concentration in the roots of *Medicago truncatula* [220]. Colonization of *Lolium perenne* by a fungal endophyte, *Neotyphodium lolii* decreased the nitrate content and several amino acids of the host plants [221].

Organic acids moderated the bacterial colonization on plant roots and increased the formation of biofilm of the root microbiome. Fumaric acid induced biofilm formation and malic acid induced chemotactic response. Organic acids from rice root exudates contain amino acid residues such as alanine, glycine, histidine, proline, and valine and carbohydrates arabinose, galactose, glucose, glucuronic acid, and mannose induced chemotactic response by endophytic bacteria *Corynebacterium flavescens* and *Bacillus pumillus* [222]. Endophytic bacteria *Pseudomonas pseudoalcaligenes* caused accumulation of higher concentrations of glycine betain-like compounds that assist in the development of tolerance to saline stress in rice [223]. Root endophytic fungus *Colletotrichum tofieldia* enhanced phosphate translocation in the colonized *Arabidopsis* plant roots in phosphate deficient conditions. Plant phosphate starvation responses (PSRs) showed regulation of the activities of the fungi. PGP activities of *C. tofieldia* were connected to pathways of the host plant immune system, indicating the active role of the plant through PSRs and the plant immune system while regulating root microbiome under phosphate stress conditions [224]. 

### 6.2. Metagenomes of Root-Associated Endophytes

With recent development in sequencing technologies, endophytic bacteria are now being identified and characterized by culture-independent methods, as only a few of these are culturable [178]. Therefore, culture-independent methods such as metagenomic analysis have been employed for the analysis of plant microbiome composition and functional genes in an environment [225]. The plant-associated endophytic community has a role in protecting the host from biotic and abiotic stress, plant growth promotion, biocontrol, nutrition, and niche adaptation, as revealed by exploring gene expressions, in correlation to genomic and proteomic analyses. Analysis of root-associated microbiome in healthy and nematode infected tomatoes showed nematode infected tomato roots resulted in a reduced abundance of predominant endophytes Streptomycetaceae and Pseudomonadales, which are known to produce active compounds against plant pathogens. Root gall-associated microbiome was found to enrich Flavobacteriales, Sphingobacteriales, and nematode-associated bacteria such as Enterobacteriales and Rhodocyclales [226]. Identification of diverse endophytic communities could lead to an understanding of their interactions, which would assist in managing crop development and health. Characterization of sorghum-associated endophytic bacterial communities showed that root and stem had diverse microbiome, however, both communities were dominated by plant growth-promoting bacterial genera *Agrobacterium*, *Erwinia*, *Herbaspirillum*, *Microbacterium*, *Pseudomonas*, *Sphingobacterium*, and *Stenotrophomonas* [227]. Inoculation of beneficial microbe caused shifts in the endophytic community structure, which resulted in increased resistance to pathogens in potato, pine, and tomato [228]. Metagenomic analysis of bacterial endophytes in rice roots revealed members of phylum Proteobacteria dominated the endophyte community. Enterobacter-related endophytes of Gammaproteobacteria and Alphaproteobacteria were found to be abundant. Several gene clusters including protein secretory systems for translocation across cytoplasmic and outer membranes were detected, which could reflect the bacterial community to the endorhizosphere as an exclusive microhabitat. Additionally, endophytic isolates encoded gene components for type VI secretion systems, which are suggestive of beneficial plant-microbe interaction. Rice endophytic microbiome was observed to consist of diverse genes related to hydrolytic plant-polymer-degrading enzymes, detoxification of reactive oxygen species (ROS), glutathione synthases, and glutathione-*S*-transferases (GST), autoinducer molecules, and iron acquisition [229]. Illumina-based 16 rRNA analysis of bacterial community structure of rhizosphere, phyllosphere, and endosphere of tomato plants showed that bacterial richness decreased from root zone soil to rhizosphere to phyllosphere to endosphere, whereas diversity was decreased from root zone soil to rhizosphere to endosphere to phyllosphere [230]. Proteobacteria was found to be the most abundant phyla associated with tomato plants. At the genus level, endophytes belonging to *Acinetobacter*, *Enterobacter*, and *Pseudomonas* were abundant in roots, leaves, and stems. Root endophytes, which had beneficial effects on plant growth, were more diverse, which was suggested to be due to the interaction between plants and soil [231]. Bacterial community structure depends on the soil type; moreover, organic soils have high moisture retaining capacity as well as organic carbon and nutrients. Endophyte communities in roots were observed to be more diverse in tobacco from organic soils when compared to mineral soils [232]. Under different soil and climatic conditions, the endophytic bacterial community structure of sweet potato was observed to be similar, which indicated that soil and climatic conditions did not affect the endophytic community. The distribution of endophytic genera was found to be dominated by *Pseudomonas,* followed by *Enterobacteriaceae-g, Erwinia, and Burkholderia* [233]. 

Metagenomics and network inference revealed that fungal infection of plant roots increased the members of *Chitinophagaceae* and *Flavobacteriaceae* in the root endosphere. This resulted in stimulating enzymatic activities related to fungal cell-wall degradation and enhanced secondary metabolite biosynthetic gene clusters encoded by NRPSs and PKSs. Further, an endophyte identified as *Flavobacterium* BGC298 genome was observed to encode a metabolite that has antifungal activity or functions such as plant protective traits [234]. The genome of endophytic *Candidatus Burkholderia* kirkii was observed to encode numerous biosynthetic genes for the production of secondary metabolites and protect its host plant *Psychotria kirkii*. The bacterium was found to produce an antimicrobial compound, C7N aminocyclitol, which showed its importance for the symbiotic association in the leaf nodule [235].

### 6.3. Proteome Analysis for the Effect of Endophytes on Host Plants

The proteomic analysis could identify modifications induced by an endophyte in plant protein expression, such as defense response and hormone production, which could alter plant-endophyte interaction. Metaproteomics determine the functional expression of the microbial community and their metabolic activities using mass spectrophotometry (MS) to characterize protein expression in a given micro-environment [236]. Proteome analysis of *Kosakonia radicincitans* DSM 16656 inoculated *Arabidopsis thaliana* revealed 12 protein spots responsive to cellular stress reactions, and inoculation of DSM 16656 increased the expression of 20S proteasome alpha-3-subunit. Endophytic colonization was inhibited by the accumulation of ubiquitin-dependent proteins and their degradation and as a result, it showed a decline in proteasome activity, however, inoculation *A. thaliana* mutant (rpn12a) defective in 26S proteasome was found to enhance the plant growth [237]. Inoculation of *Sinorhizobium meliloti* in rice showed upregulation of plant defense-related proteins in roots and photosynthesis-related proteins in aerial tissues [238]. Similarly, Lery et al. [239], demonstrated differences in the expression of defense response and signaling-related protein, which resulted in different colonization between two sugarcane cultivars with the inoculation of *Gluconacetobacter diazotrophicus*. The proteomic approach to study plant response towards colonization with endophyte *Azoarcus* sp. strain BH72 showed that jasmonic acid (JA) could restrict endophytic colonization, indicating that plant defense could control the entry of the endophyte. Rice (*Oryza sativa*) variety cv. IR42 showed less interaction with the endophyte with JA-induced proteins while other variety *O. sativa* cv. IR36 was colonized by the bacterium induced by JA, which was also induced by the endophyte (SalT, two isoforms). Seven JA-induced proteins were also observed to be induced by the bacterium in cv. IR42 [240]. Fungal endophyte *Gilmaniella* sp. AL12 inoculation in *Atractylodes lancea* resulted in a 2.7% differential gene expression. The upregulated genes were having functional roles in both, primary and secondary metabolism, along with proteins for terpene skeleton biosynthesis. Genes involved in cytokinin biosynthesis and signal transduction were upregulated, which induces cell division and enhanced chlorophyll biosynthesis [241].

### 6.4. PGPR Impact on the Plant Transcriptome

Root inoculation by suitable bacteria, including endophytes, and its effect on different physiological functions of the plant has been described. In *Arabidopsis* leaves, the overexpression of a total of 520 genes and suppression of a total of 364 genes (threefold changes) was initiated by *P. putida* MTCC5279 inoculation; upregulated genes were mainly those that have functions similar to genome integrity preservation, repression of ethylene and ABA signaling, and ISR induction and Ca^2+^ mediated signaling of ISR [242]. *Azospirillum brasilense* Sp245 inoculation was followed by the expression of ethylene receptors in two cultivars of rice that had the distinct abilities to acquire nitrogen [243]. In another interesting study, the application of endophyte, *Azoarcus* resulted in the accumulation of transcripts for ethylene receptors in rice plants. Similarly, inoculation with endophytic PGPR *Herbaspirillum seropedicae* also resulted in altered expression of ethylene, auxin, and defense-linked proteins in rice roots [244]. These indicated that during colonization, the plant defense responses are modulated by endophytes. Inoculation of *Pseudomonas fluorescens* WCS417r, in *Arabidopsis* plants, 97 genes within the roots had a different expression, while in leaves, not even a single gene (among 8000 genes) had any significant change in expression level [245]. The transcriptome data also revealed that ISR is induced by *P. fluorescens* SS101 via salicylic acid signaling in the host plants [246]. In wheat, the accumulation of defense-associated transcripts was influenced by the bacterization with *P. fluorescens* Q8r1-96, while the number of transcripts for type three secretion systems and DAPG was not affected [247].

## 7. Conclusions

Root endophytes benefit the host plants directly or indirectly by producing essential phytohormones such as IAA, siderophore, abscisic acid, cytokinin, and solubilize phosphates, which stimulate the growth of the plants. Root endophytes interact with plant roots and assist in nutrient exchange. In addition, the root endophytic microbiomes effectively induce plant defense with quorum sensing response against viral and bacterial pathogens. Advances in emerging technologies such as transcriptomics and metabolomics have provided insights into colonization of the rhizosphere by plant-endophyte and their interaction, which could develop new strategies to improve tolerance to stress. A deeper insight into the role of the root endophytic microbiome for improving plant growth and the physiology of the host plants needs to be explored, along with other factors that shape the specific rhizo-microbiome. This could mark the potential use of endophytes for the benefit of plants and enhance yield. Therefore, it is important to study the diversity of root endophytic microbiomes, their colonization, and their activity for successful application in agricultural lands. 

## Figures and Tables

**Table 1:** Association of endophytes with respective host plants and their beneficial attributes for plant growth promotion.

Sl. No.	Endophyte	Host-Plant	PGP-Attributes	Reference
1.	*Paenibacillus polymyxa* SK1	*Lilium lancifolium*	1-Aminocycloprapane-1-carboxylic acid (ACC), deaminase, indole-3-acetic acid (IAA), siderophores, nitrogen fixation, and phosphate solubilisation, showed antifungal activities against plant pathogens	[46]
2.	*Paenibacillus glycanilyticus* LJ121 and *Pseudomonas* *brenneri* LJ215	Lupine root	Increase shoot Dry weight, number of nodules per plant, photosynthetic assimilation rate and chlorophyll a and b content and shoot nitrogen and phosphorus content	[47]
3.	*Arthrobacter* sp. EpS/L16	*Echinacea purpurea*	IAA production, increase in the number of leaves	[48]
4.	*Lysinibacillus* sp. S24 *Brevibacterium* sp. S91	Tea (*Camellia sinensis* L.)	Highest phosphate solubilisation, IAA and ammonia production	[49]
5.	*Enterobacter cloacae* R7	Maize roots	IAA (35.4 mg mL_1), ACC deaminase (+), siderophore (+), and phosphate solubilization (+), alleviating heavy metal stress	[50]
6.	*Bacillus cereus* N5	Maize roots	IAA (47.3 mg mL_1), ACC deaminase (+), siderophore (+), and phosphate solubilization (+), tolerance of this plant to environmental stresses	[50]
7.	*Streptomyces exfoliatus* FT05W	Lettuce roots	solubilize phosphates and to synthesize IAA, active against other soil borne fungal pathogens	[51]
8.	*Stenotrophomonas rhizophila* ep-17	Soybean	Beneficial association with *Bradyrhizobium* in the rhizosphere and promote plant growth, nutrient uptake and grown soybean under salt stress condition.	[52]
9.	*Bacillus subtilis* SU47 and *Arthrobacter* sp. SU18	Wheat	Showed an increase in dry biomass, total soluble sugars and proline content	[53]
10.	*Bacillus pumilus* 2-1, *Chryseobacterium indologene* 2-2, and *Acinetobacter johnsonii* 3-1	Sugar beet	Increased photosynthetic capacity, increased concentration of carbohydrates	[54]
11.	*Burkholderia phytofirmans* strain PsJN	Potato, tomato, Onion, maize, barley	ACC deaminase activity, IAA synthesis	[55]
12.	*Pseudomonas syringae*	*Arabidopsis* *thaliana*	IAA and abscisic acid biosynthesis	[56]
13.	*Gluconacetobacter diazotrophicus*	Sugarcane	Nitrogen fixation	[57]
14.	*Rhizobium leguminosarum* bv. trifolii	Rice roots	Biological N_2_ fixation	[58]

## Data Availability

Not applicable.
